# Spectral imaging and analysis of monophasic CT angiography to assess infarct core and penumbra in acute stroke

**DOI:** 10.1038/s41598-024-78789-2

**Published:** 2024-11-18

**Authors:** Schekeb Aludin, Lars-Patrick Schmill, Patrick Langguth, Olav Jansen, Naomi Larsen, Fritz Wodarg, Tristan Klintz, Svea Seehafer, Agreen Horr

**Affiliations:** Department of Radiology and Neuroradiology, University Hospital Schleswig-Holstein, Campus Kiel Arnold-Heller-Str. 3, Haus C/D, D-24105 Kiel, Germany

**Keywords:** Stroke, Stroke

## Abstract

Acute stroke imaging includes native CT, CT-angiography (CTA), and CT-perfusion (CTP). CTP assesses the irreversibly damaged infarct core (IC), and the potentially salvageable penumbra (PEN) and distinguishes these from areas of healthy parenchyma (HA). However, it requires additional contrast agent and radiation. Spectral-CT (SCT) enables spectral imaging like e.g., iodine-density imaging, and we evaluated its potential in estimating IC and PEN using monophasic CTA data only. We analysed 28 patients with mediainfarction. CTP-analysis derived areas of IC, PEN and HA on infarction side, as well as their healthy hemisphere’s counterparts were transferred to CTA as Region of interest (ROI). Spectral measurements included Hounsfield-Units in monoenergetic maps (MonoE) at 40 keV, 70 keV, and 120 keV, plus iodine-density (ID) and electron-density (ED) values, totalling 2970 values. Unilateral absolute values and ratios to the healthy counterparts were evaluated. Visual infarct delineation on each map was also rated. In all spectral maps, the infarct areas could be distinguished from the healthy counterpart by absolute values (*p* < 0.05). IC, PEN and HA could be distinguished from each other by absolute values (*p* < 0.05) (except for ED), and by the ratio-value formed to the contralateral side (*p* < 0.05). Detection of IC and PEN were best possible in ID (IC (AUC = 0.9999, *p* < 0.0001); PEN (AUC = 0.9745, *p* < 0.0001)) and MonoE40 (IC (AUC = 0.9963, *p* < 0.0001); PEN (AUC = 0.9622, *p* < 0.0001)). Differentiation of IC and PEN was also best in ID (AUC = 0.93, *p* < 0.0001) and MonoE40 (AUC = 0.80, *p* < 0.0001). Similarly, visual delineation was best too in ID and MonoE40. Accordingly, IC and PEN can be detected and differentiated in monophasic CTA by using SCT-derived spectral maps like ID or MonoE40.

## Introduction

Multimodal cerebral computed tomography (CT) imaging is the most important diagnostic modality in acute ischemic stroke (AIS)^1^. Apart from native cranial CT (NCCT) and CT angiography (CTA), which are used to detect early ischemic signs or vascular occlusion, CT perfusion (CTP) assesses cerebral perfusion^1,2^. It detects hypoperfusion and quantifies the irreversibly damaged infarct core (IC) and the potentially salvageable penumbra (PEN), as an important criterion for therapeutic decisions such as thrombolysis or thrombectomy^3–5^. However, despite its crucial clinical role, CTP also involves a second administration of iodinated contrast and additional radiation exposure^3,4^. Dual-layer spectral detector CT (SDCT) is a new technique of spectral computed tomography (SCT), which enables registration of material-specific and energy-dependent attenuation behaviours in every scan without prior protocol administration^6,7^. From acquired spectral data, material-specific or virtual monoenergetic (MonoE) maps can be created. E.g., iodine density (ID) maps can be used to determine the iodine concentration in tissues and to assess perfusion defects, as already being clinically evaluated and used in the lung or abdominal organs^7–11^. In this way, tissue perfusion can be quantified after administering a single dose of contrast agent.

We investigated whether spectral analysis of monophasic CTA performed in multimodal AIS imaging can be used to reliably quantify and assess cerebral perfusion to detect and identify IC and PEN in comparison to CTP.

## Methods

### Study design and cohort

This retrospective study was approved by the local IRB of the Medical Faculty of the Christian-Albrechts-University of Kiel. The research involved in this study was conducted in accordance with relevant guidelines and regulations, and the declaration of Helsinki. Informed consent was obtained from all patients. We analysed patients who were admitted to the emergency department of our hospital (a tertiary hospital with a stroke unit) between March 2020 and February 2022 with proven AIS in the media territory and in whom interventional therapy was indicated owing to a relevant PEN/IC mismatch. Inclusion criteria: (1) imaging by multimodal CT (NCCT, CTA, and CTP) with an SDCT; (2) available spectral datasets and CTP datasets; (3) confirmed large-vessel occlusion affecting the media territory (internal carotid artery (ICA) or middle cerebral artery on level M1 or M2); (4) absence of higher-graded (> 70%) stenosis in the contralateral common carotid artery (CCA) or the contralateral internal carotid artery (ICA). Exclusion criteria: (1) artefacts in the CTP dataset due to motion or poor contrast (automatically indicated by the CTP analysis software used in this study, IntelliSpace Portal^®^ (ISP) (IntelliSpace Portal^®^ V.10, Philips Healthcare, Best, the Netherlands)); (2) presence of hemorrhage, tumors or postischemic lesions in the media territory or embolism in other cerebrovascular territories; (3) in order to clearly estimate and differentiate between IC and PEN, only patients who displayed both a PEN and an IC on semi-automated CTP analysis and additionally an IC volume of at least 10 ml were included; (4) missing consent.

### Imaging by SDCT

All CT examinations were performed on a 128-section SDCT (IQon, Philips Healthcare, Best, the Netherlands). CTA began at the level of the ascending aorta and covered the entire neck and head region. The acquisition parameters of CTA are given in Table 1. Conventional images were reconstructed at 1-mm slice thickness and the following spectral maps were obtained: ID, electron density (ED), and MonoE at 40 keV (MonoE40), 70 keV (MonoE70) and 120 keV (MonoE120). Spectral maps were analysed using ISP.

CTP required a second application of contrast agent, and the scan covered the whole brain parenchyma by using the toggling-table technique. The acquisition parameters for CTP are given in Table 1. Eight slices of CTP with a thickness of 10 mm were reconstructed in each patient.

**Table 1:** Technical scan parameters.

Technical parameters	CT angiography	CT perfusion
Tube voltage	120 kV	120 kV
Pitch	1.046	1.046
Gantry rotation time	0.4 s	0.4 s
Scan time	4.4 s	58 s
Collimation	64 × 0.625	64 × 0.625
Scan delay	4 s	5 s
Volume of contrast agent	60 ml	40 ml
Infusion speed	5 ml/s	4 ml/s
Number of cycles	–	17

### Image analysis

**Fig. 1:**
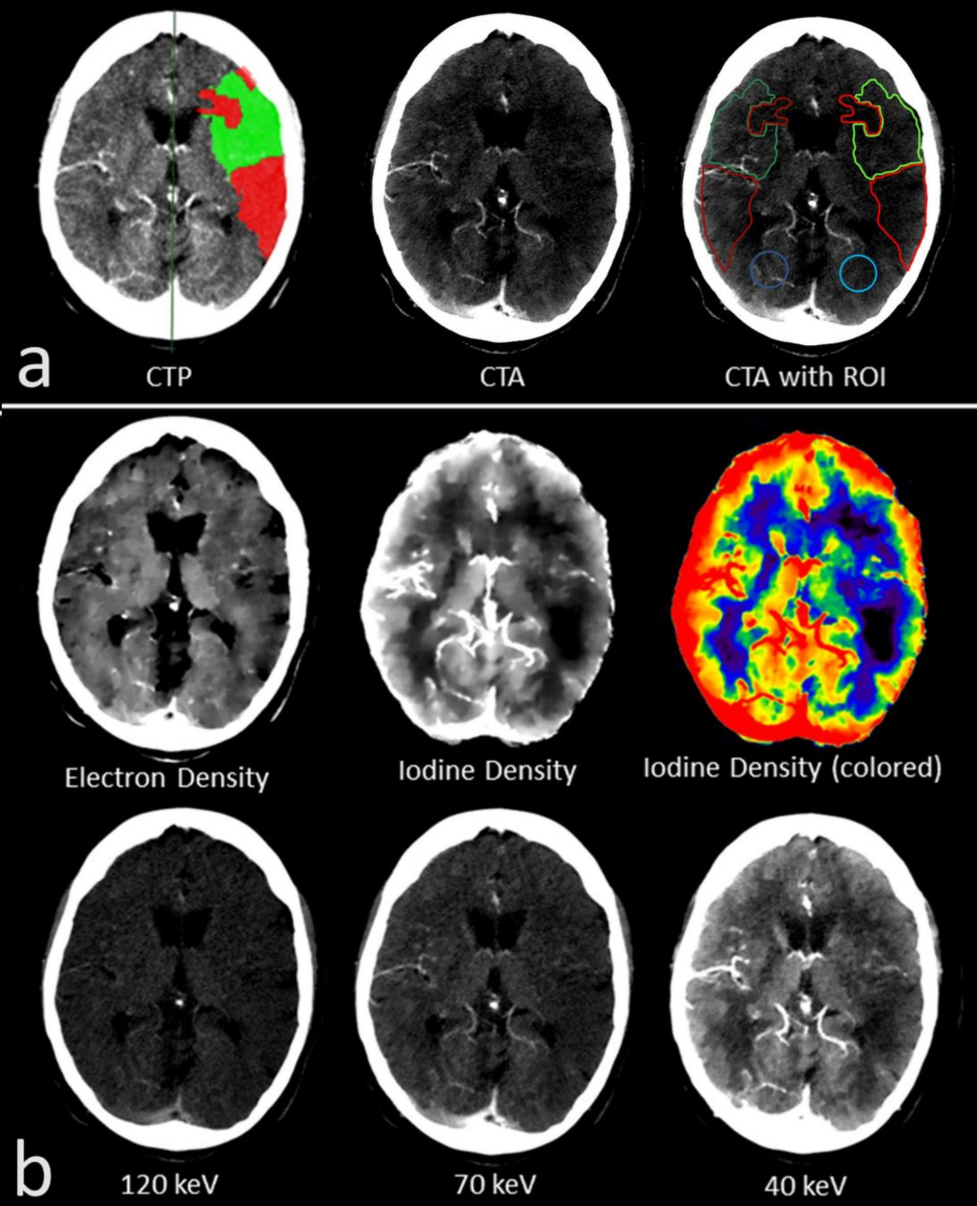
(**a**) The semi-automated computed tomographic perfusion (CTP) analysis (left) indicating the infarct core (IC; red area) and the penumbra (PEN; green area) in an individual with M1 occlusion on the left hemisphere. IC and PEN were transferred and outlined as regions of interest (ROIs) to the corresponding plane in the computed tomographic angiography (CTA; right) (IC = light red; PEN = light green). An area of healthy brain parenchyma on the affected side was defined (HA = light blue). Additionally, the ROIs were mirrored to the contralateral healthy side for comparison (mirrored IC = dark red; mirrored PEN = dark green; mirrored HA = dark blue). (**b**) Representation of the different spectral maps in the same patient with M1 occlusion on the left hemisphere. In the spectral maps, the infarct area can be assessed well in the iodine density (ID) and the MonoE 40 keV map. Visual assessment was best in the ID map, which is therefore displayed as gray/white and additionally in color.

All examinations were analysed by two radiologists with several years of experience in acute stroke imaging (S.A., A.H.) and who were blinded to any clinical data other than from the infarct side. First, semi-automated perfusion analysis of the CTP was performed by applying literature-based parameters for IC and PEN^12^. The areas of sole IC and sole PEN were indicated by the software (Fig. 1a), but as an IC and a PEN were not present in all eight slices across the whole brain, only those slices given both, IC and PEN, were used for further analysis.

#### Quantitative assessment

To investigate the quantitative detection and differentiability of the IC and PEN regions, spectral parameters of these areas were measured in the different spectral maps. The infarct areas of IC and PEN given in the CTP analysis were outlined and transferred manually to the corresponding layer of the CTA as region of interest (ROI) (Fig. 1a, reconstruction at 10-mm slice thickness as set for the CTP). Parts that were indicated by the analysis but obviously did not correspond to the infarct area (e.g., large parts of the bone or cerebrospinal fluid) or contained larger vessels were omitted from the measurement whenever possible. In addition to the IC and PEN areas, a healthy area (HA) was also recorded on the infarct-affected hemisphere, which was intended to differentiate IC and PEN from healthy brain tissue. For this purpose, a ROI of at least 200 mm² was drawn in the brain parenchyma of the posterior or anterior flow territory, which was not affected by the infarction and care was taken to ensure that there was no evidence of involvement present in the CTA and according to CTP analysis. In addition to these areas which were measured on the infarct side (IC, PEN and HA), corresponding areas were also mirrored on the healthy hemisphere and marked as ROI (IC*, PEN*, HA*) (Fig. 1a). For quantitative assessment, the spectral values for IC, PEN and HA as wells as for IC*, PEN* and HA* were measured from the respective ROIs in the different spectral maps (mg/ml for ID, percentage-values for ED and Hounsfield-unit (HU) for MonoE). In addition to comparing the measured absolute values of the different areas, bilateral ratios were also calculated from these values for comparison. Therefore, the following ratios between IC, PEN, HA and their healthy counterparts were calculated from the measured spectral values in each spectral map: (IC/IC*), (PEN/PEN*) and (HA/HA*).

#### Visual assessment

The spectral maps were also rated for visual assessment of the infarct dimension. This visual assessment was performed by the raters in a separate reading session held at least four weeks apart from the reading session for quantitative assessment. As described above, the areas for PEN and IC were highlighted by the semi-automated CTP analysis in the CTP-slices. These highlighted CTP-slices were used as a reference and compared with the corresponding slices of the CTA in the different spectral maps (Fig. 1b). The visual delineation of the infarct area in each spectral map was rated using a 5-point Likert scale (1 = no delineation, 2 = poor delineation, 3 = moderate delineation, 4 = good delineation, and 5 = very good delineation). The raters were allowed to flexibly change the windowing of the images. The different spectral maps were visually rated in different sessions to avoid bias.

### Statistical analysis

Statistical analysis was performed using GraphPad Prism 9 (GraphPad Software, Boston, USA). Normal distribution was tested with the D’Agostino & Pearson test for all values. Measurements for the different spectral maps were compared with a 2-way ANOVA and Tukey’s multiple comparison test. Depending on the distribution, comparison of ratios was done with a repeated-measures one-way ANOVA and Tukey’s multiple comparison test or by using the Friedman test and Dunn’s multiple comparison test. Interrater correlation was analysed with Kendall-Tau or Pearson correlation coefficient, depending on the scale level. Furthermore, Receiver-Operating-Curve (ROC) analysis and Area-Under-the-Curve (AUC) calculation was performed for the different spectral parameters. Youden-Index was used for the calculation of cut-off values.

## Results

### Study cohort

**Fig. 2:**
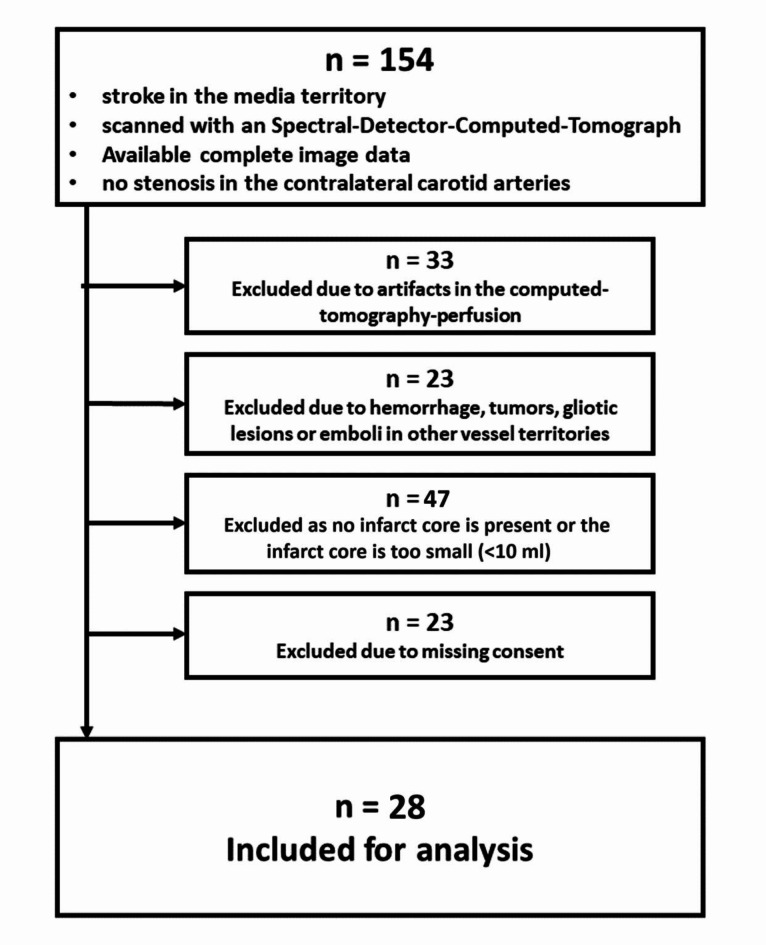
Retrospective evaluation of the study cohort with inclusion/exclusion criteria.

The retrospective evaluation identified 154 patients who had suffered a stroke in the media territory and received multimodal CT imaging in an SDCT with acquired SBI-dataset. Ultimately, 28 patients met the inclusion/exclusion criteria (Fig. 2). Basic demographic and clinical data are given in Table 2.

**Table 2:** Characteristics of the study cohort.

Parameters	Values
Age (years)	79.4 (43–90)
Sex	
Men	12
Women	16
Occluded vessel-segment	
Internal carotid artery	7
Middle cerebral artery M1-segment	14
Middle cerebral artery M2-segment	7
Alberta-Stroke-Program-Early-CT-Score	7.67 ± 2.24
Volume of infarction core (ml)	49 ± 47.11
Volume of penumbra (ml)	145.37 ± 48.75
Ratio (volume of penumbra / volume of infarction core)	4.8 ± 2.7

Unless otherwise indicated, data are mean ± standard deviation, ranges in parentheses.

### Quantitative assessment

In total, an IC and PEN were present in 99/224 CT slices. Accordingly, 594 areas (IC, PEN, HA, IC*, PEN* and HA*; 99 of each) and a total of 2970 measurements were analysed. Interrater reliability was excellent (*r* = 0.92).

**Fig. 3:**
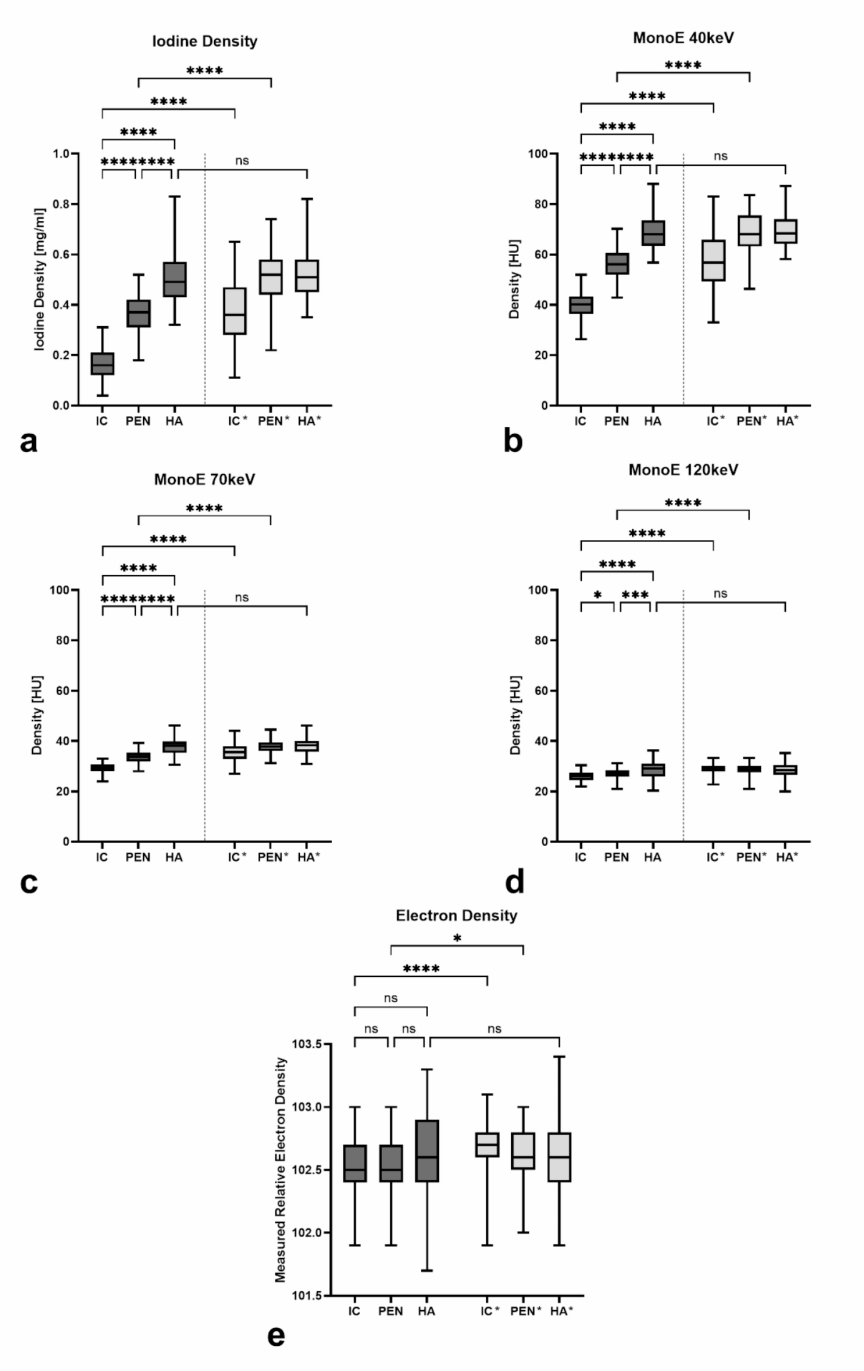
Representation of the absolute values measured for the different areas on the infarcted side (IC = infarct core; PEN = penumbra; HA = healthy area) and on the healthy opposite side (IC*, PEN*, HA*) with respect to each spectral parameter. Statistical testing was done to compare IC, PEN and HA between each other as well as to the contralateral regions. Boxes show the median with the range from the 25th percentile to the 75th percentile. Whiskers show the range from minimal to maximal values. (**a**) iodine density; (**b**) MonoE 40 keV; (**c**) MonoE 70 keV; (**d**) MonoE 120 keV; (**e**) electron density. *ns* not significant. *: *p* < 0.05; **: *p* < 0.005; ***: *p* < 0.0005; ****: *p* < 0.0001.

A comparison of the measured absolute values in the different spectral maps is shown in Fig. 3. The comparison of the areas on the infarcted side shows that the absolute values of IC are the lowest, followed by PEN and finally HA. The statistical comparison shows a significant differentiability for IC and PEN compared to HA in all spectral maps (*p* < 0.0005), except for ED (*p* > 0.05). The comparison between IC and PEN also shows a significant differentiability between them in all spectral maps, except for ED. Here, differentiation between IC and PEN was best for ID, MonoE40, and MonoE70 (each *p* < 0.0001) and could also be distinguished in MonoE120 (*p* < 0.05). In contrast, IC and PEN cannot be significantly distinguished from each other in ED. In addition to the comparison of the values on the infarct-affected side, Fig. 3 also shows the comparison of the IC, PEN and HA areas with their contralateral, mirrored area. In all spectral maps, there is a significant differentiability of IC compared to IC* and PEN compared to PEN*, with the values on the infarct-affected side being lower (at least *p* < 0.05). In contrast, HA is not significantly differing from HA* in any of the spectral maps.

**Fig. 4:**
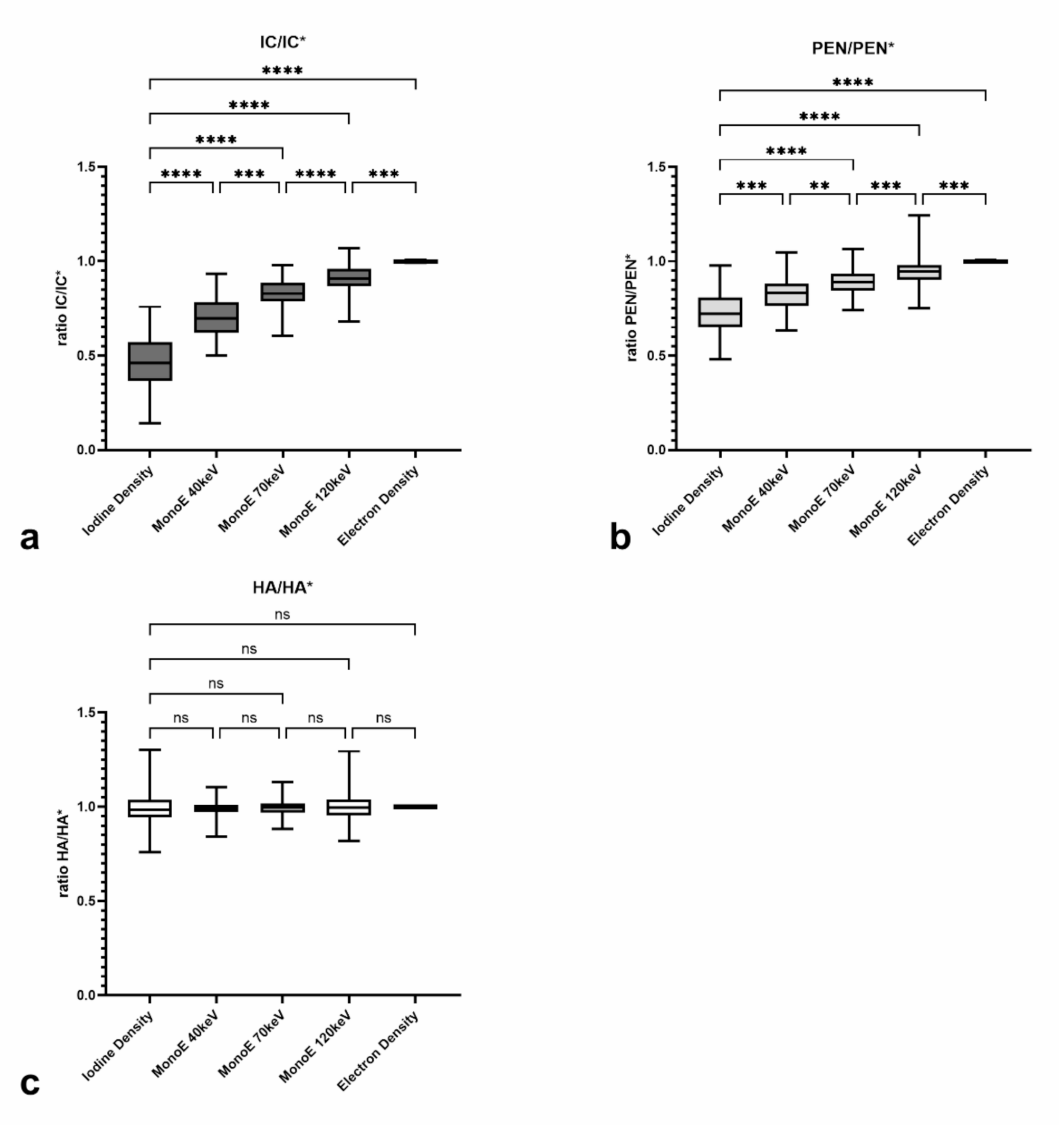
(**a**) Ratio between the infarct core (IC) and the corresponding contralateral region IC* (IC/IC*) for each spectral parameter; (**b**) Ratio between the penumbra (PEN) and the corresponding contralateral region PEN* (PEN/PEN*) for each spectral parameter; (**c**) Ratio between the healthy area (HA) and the corresponding contralateral region HA* (HA/HA*) for each spectral parameter. Statistical comparison was done between the iodine density and every other spectral parameter as well as between the parameters that follow each other in the ratio-values. Boxes show the median with the range from the 25th percentile to the 75th percentile. Whiskers show the range from minimal to maximal values. *ns* not significant. *: *p* < 0.05; **: *p* < 0.005; ***: *p* < 0.0005; ****: *p* < 0.0001.\.

**Fig. 5:**
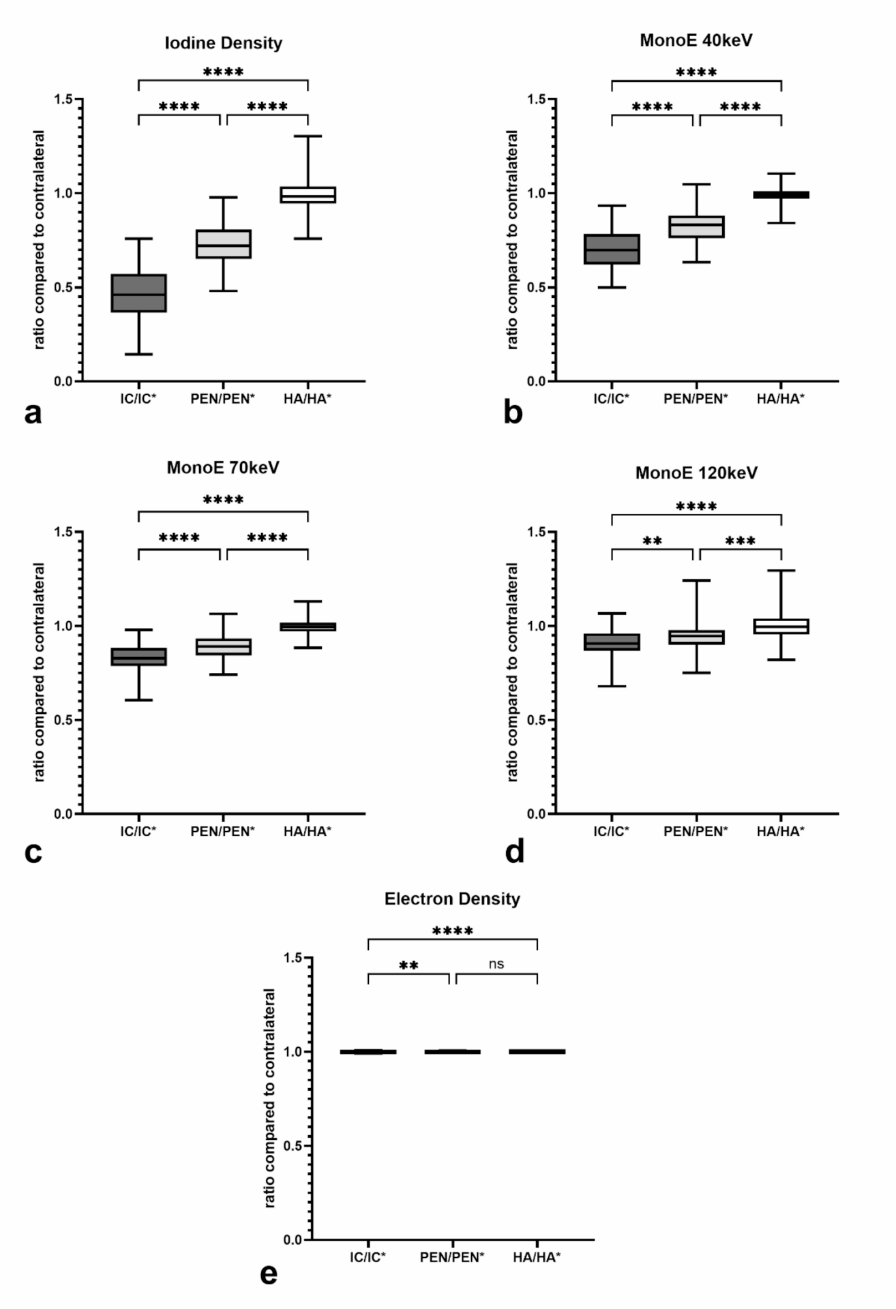
(**a**–**e**) Ratio of infarct core (IC), penumbra (PEN) and healthy area (HA) versus their healthy counterparts (IC*, PEN* and HA*) with respect to each individual spectral parameter. (**a**) iodine density; (**b**) MonoE 40 keV; (**c**) MonoE 70 keV; (**d**) MonoE 120 keV; (**e**) electron density. Statistical testing was done for the comparison between ratios for the infarct core (IC/IC*), the penumbra (PEN/PEN*) and the healthy area (HA/HA*). Boxes show the median with the range from the 25th percentile to the 75th percentile. Whiskers show the range from minimal to maximal values. *ns* not significant. *: *p* < 0.05; **: *p* < 0.005; ***: *p* < 0.0005; ****: *p* < 0.0001.

**Fig. 6:**
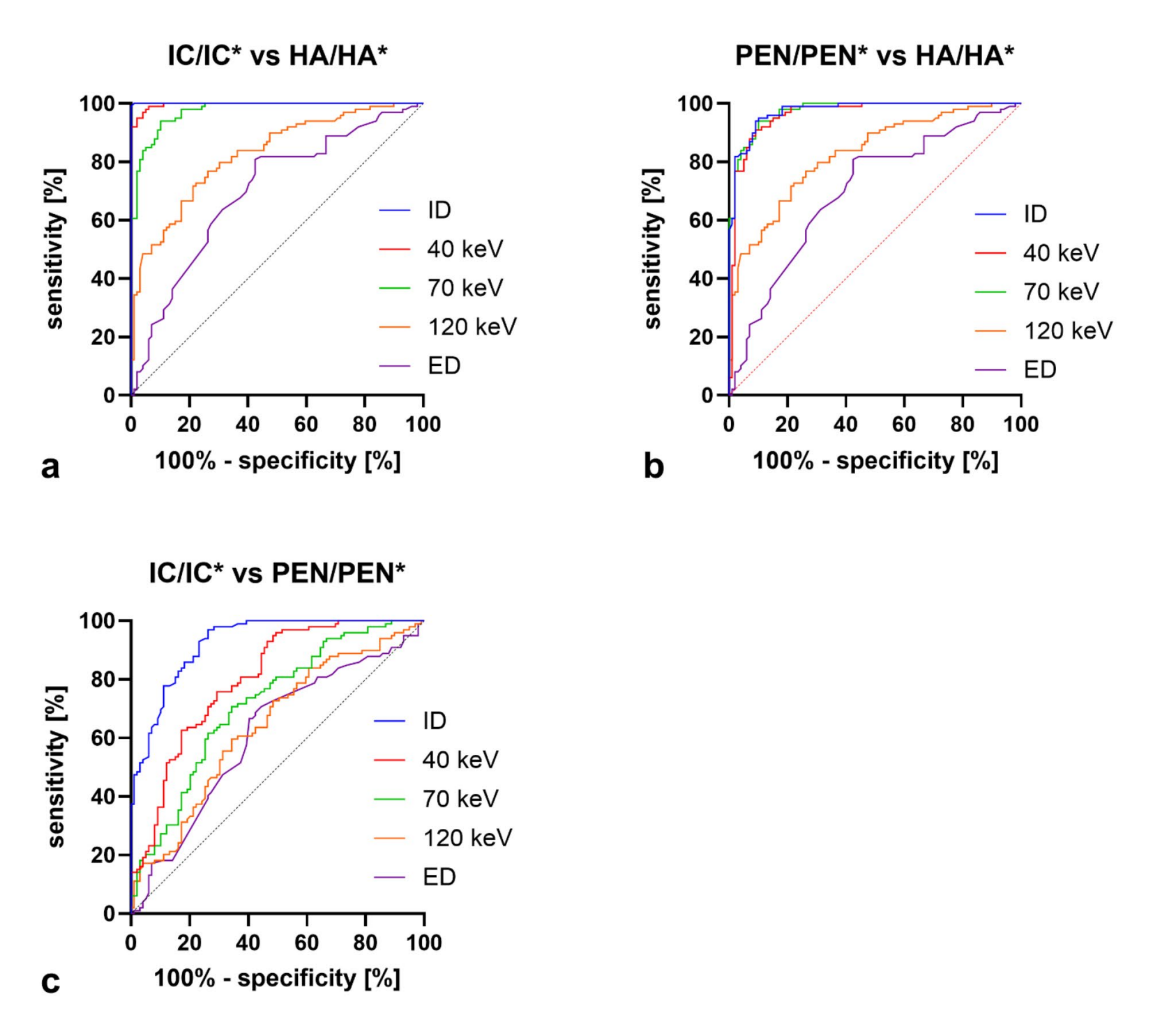
(**a**) Receiver-Operating-Curve for differentiation of the infarct core and the healthy area based on the ratios IC/IC* and HA/HA* for each spectral map. (**b**) Receiver-Operating-Curve for differentiation of the penumbra and the healthy area based on the ratios PEN/PEN* and HA/HA* for each spectral map. (**c**) Receiver-Operating-Curve for differentiation of the infarct core and the penumbra based on the ratios IC/IC* and PEN/PEN* for each spectral map.

Apart from absolute values, the ratios IC/IC*, PEN/PEN* and HA/HA* as relation of the ipsilateral spectral values compared to the contralateral values, were also investigated. The ratios are shown in Fig. 4a for IC/IC*, in Fig. 4b for PEN/PEN* and Fig. 4c for HA/HA* regarding the different spectral parameters. In IC/IC* and PEN/PEN* ID has the significantly lowest ratios compared to the other spectral parameters; thus, IC and PEN could be best distinguished from their contralateral counterpart in this spectral map, followed by MonoE40. With increasing keV, the values of the ratios also increased and, consequently, the difference to the contralateral side became less pronounced. In contrast, regarding HA/HA*, there are no significant differences between the different spectral maps, and all ratio values are close to 1. Figure 5 compares the ratio values IC/IC*, PEN/PEN* and HA/HA* for the different spectral maps. The ratio values of IC/IC* are lowest in all spectral maps, followed by PEN/PEN* and highest for HA/HA*. In a statistical comparison, the values of IC/IC* and PEN/PEN* were significantly lower than HA/HA* for all spectral maps (*p* < 0.0005), except for ED (*p* > 0.05). IC/IC* was also significantly lower than PEN/PEN* in all spectral maps (at least *p* < 0.005), with the difference being most pronounced in ID and MonoE40 (*p* < 0.0001). In Fig. 6, ROC analyses were performed with the ratio values. Figure 6a analyses the differentiability between infarct core and healthy tissue and thus the detection of the infarct core using the ratio values IC/IC* and HA/HA*. Best differentiability is given in the ID map with a cut-off ratio < 0.7429 (sensitivity = 100%, specificity = 98.99%, AUC = 0.9999 95% CI 0.9997 to 1.0, *p* < 0.0001), followed by MonoE40 (AUC = 0.9963 95% CI 0.9922 to 1.0, *p* < 0.0001). In Fig. 6, the differentiability between penumbra and healthy tissue and thus the detection of the penumbra is analysed using the ratio values PEN/PEN* and HA/HA*. The best differentiation is possible in the ID map with a cut-off ratio < 0.8892 (sensitivity = 93.93%, specificity = 90.91%, AUC = 0.9745 95% CI 0.9572 to 0.9919, *p* < 0.0001), followed by MonoE40 (AUC = 0.9622 95% CI 0.9261 to 0.9884, *p* < 0.0001). Finally, Fig. 6 analyses the differentiability between infarct core and penumbra using the ratio values IC/IC* and PEN/PEN*. Here, too, the best differentiation is possible in the ID map with a cut-off ratio < 0.5585 (sensitivity = 96.97%, specificity = 73.74%, AUC = 0.9261 95% CI 0.8921 to 0.9601, *p* < 0.0001), followed by MonoE40 (AUC = 0.8020 95% CI 0.7413 to 0.8626, *p* < 0.0001).

### Visual assessment

**Fig. 7:**
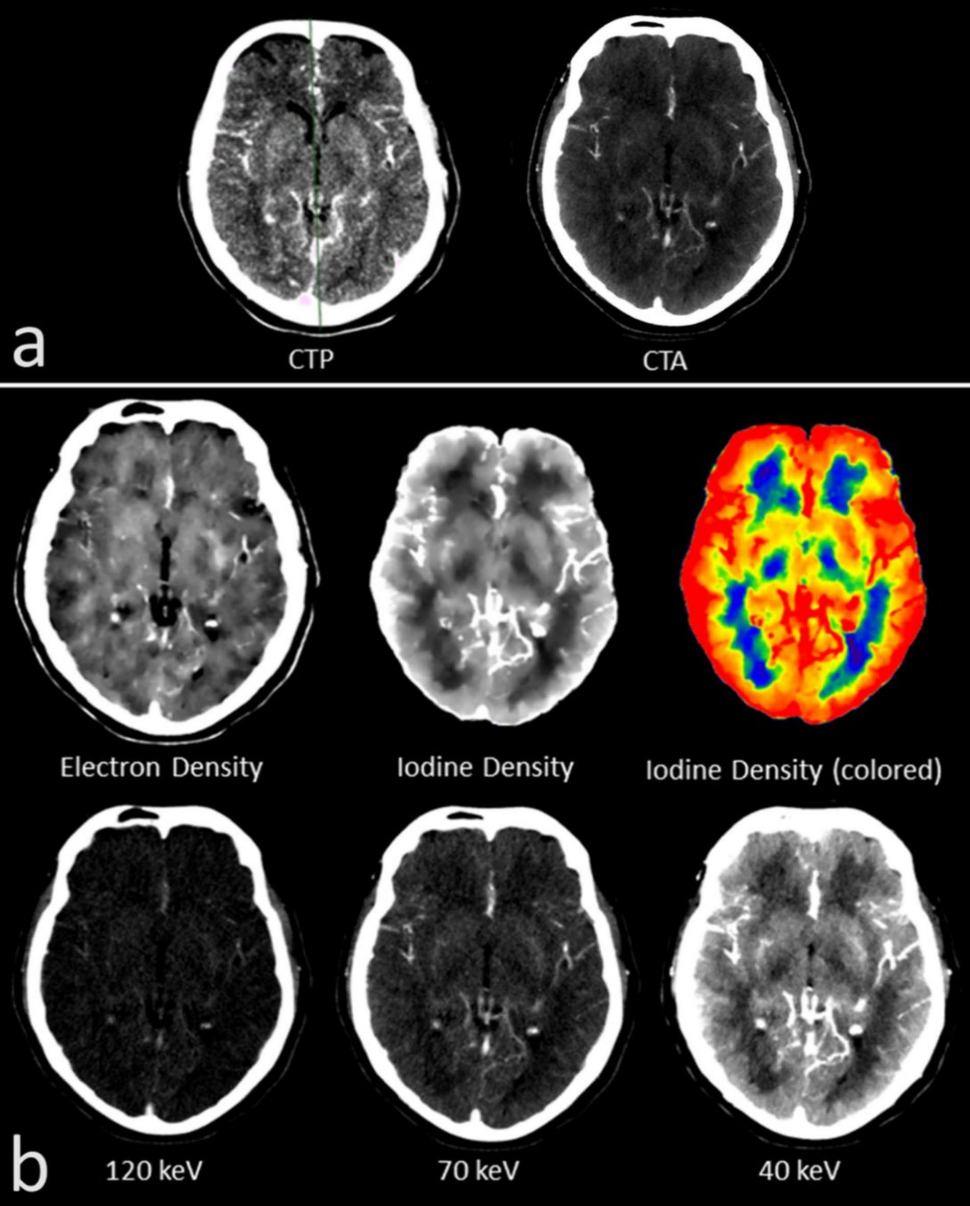
Representation of the different spectral maps in a patient without cerebral pathology. (**a**) Corresponding plane of images from computed tomographic perfusion (CTP; left) and computed tomographic angiography (CTA; right). (**b**) Different spectral maps of the same plane are depicted. Visual assessment was best in the iodine density map, which is therefore displayed as gray/white and additionally in color.

**Fig. 8:**
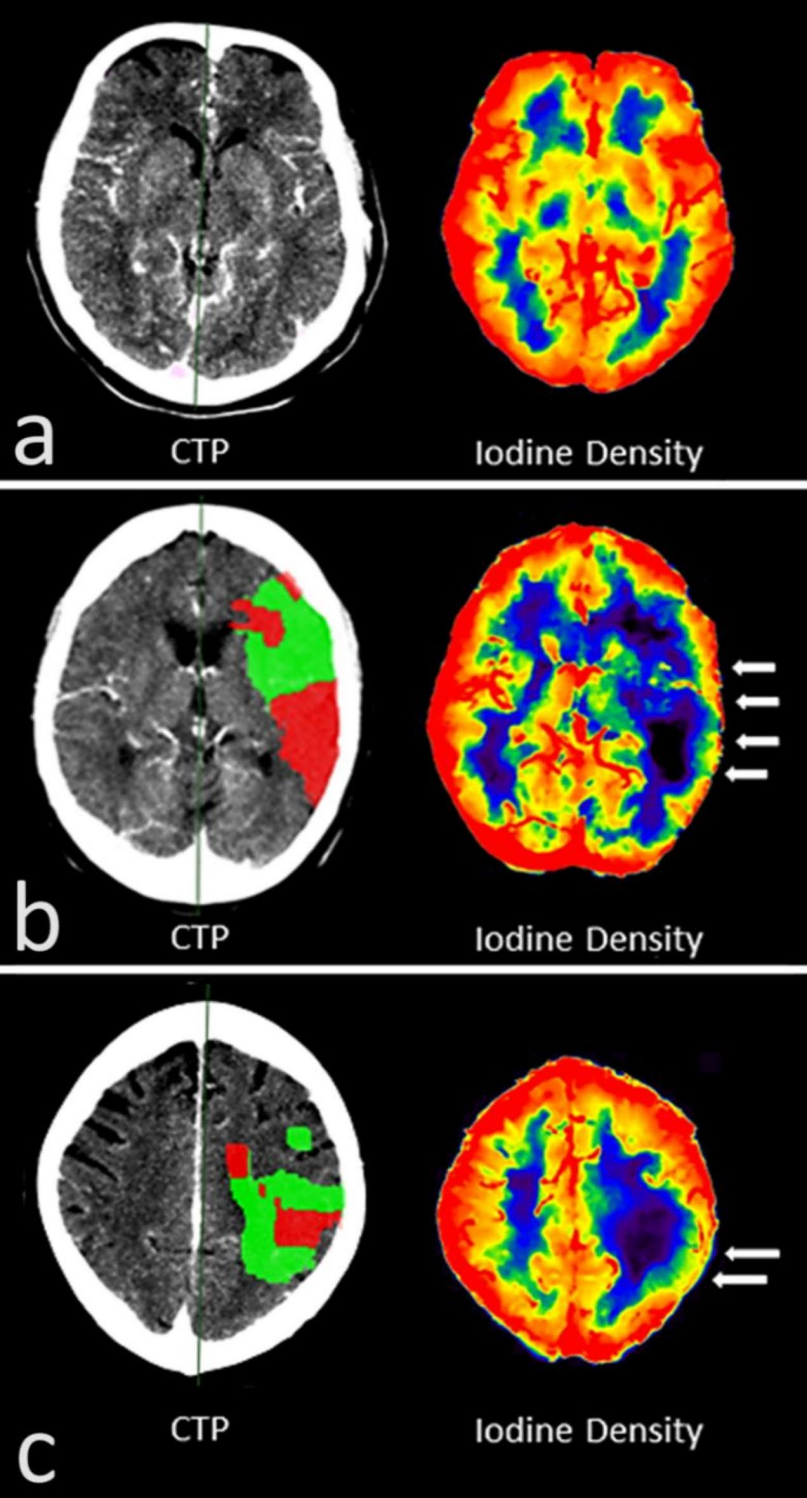
(**a**) Computed tomographic perfusion (CTP) analysis and spectrally derived iodine density (ID) map of a patient without cerebral pathology. The ID shows a homogeneous iodine accumulation in the cortical band that can be delineated peripherally as a correlate of the enhanced perfusion of the gray matter there. In addition, there is a circumscribed enhanced perfusion of the basal ganglia and a relatively lower concentration in the white matter. (**b**) CTP analysis and spectrally derived ID map of a patient with M1 occlusion on the left. The ID map shows a loss of the cortical iodine rim in the infarct area relative to the contralateral area as a pictorial correlate of the reduced perfusion (white arrows). In addition, there is also weaker perfusion in the affected medullary bed. (**c**) CTP analysis and spectrally derived ID map of a patient with M2 occlusion on the left. Like (**b**), the ID map shows a loss of the cortical iodine rim in the infarct area (white arrows) relative to the contralateral area and a weaker perfusion in the infarct-affected medullary bed.

Like the quantitative assessment, visual assessment of the infarct area was performed in the 99/224 CTP slices, that offered an IC and PEN. Interrater reliability regarding the visual assessment was good (*r* = 0.71). Visual delineation of the infarct dimension differed significantly between the different spectral maps (*p* < 0.01). Basically, cerebral infarct could be identified by the reduced contrast enhancement compared to the unaffected parenchyma (Fig. 1). In the monoenergetic maps, visual delineation of the infarct dimension became increasingly better with decreasing keV. Here, the infarct area became progressively more hypodense than the healthy parenchyma and visual delineation improved from MonoE120 (mean ± SD = 1.61 ± 0.66) to MonoE70 (2.71 ± 1.09) and up to MonoE40 (3.65 ± 1.05) (*p* < 0.0001). Although not statistically relevant, an even slightly better visual delineation than in the MonoE40 was possible in the ID map (3.76 ± 1.11), which only depicted the iodine while subtracting other materials including the skull bone. The spectral maps of an individual without cerebral pathology are given in Fig. 7 for comparison. A particularly interesting visual feature, which could only be clearly delineated in the ID map with subtracted skull bone, was the prominent contrast enhancement of the cerebral parenchyma in the cortex area. In the cortex area of the healthy tissue or hemisphere, the tissue concentration of iodine along the cortical band was always increased, giving the impression of a cortical iodine rim. In infarct-affected areas, particularly the IC, there was a loss of this iodine rim as a correlate of reduced cortical perfusion (Fig. 8). In contrast, visual delineation of the infarct dimension was barely possible in the ED map (1.60 ± 0.66).

## Discussion

In the past, studies have already been conducted in AIS patients that attempted to characterise ischemic areas by CTA alone, without CTP. Schramm et al. demonstrated that the IC could be distinguished from healthy tissue in conventional monophasic CTA datasets by correlating HU-based data from CTA with magnetic resonance imaging (MRI) perfusion analyses^13,14^. Recent studies picked up on this idea and trained artificial intelligence (AI)-based algorithms to detect the IC by conventional monophasic CTA. These algorithms were used to detect the difference in HU between vital parenchyma and ischemic areas resulting from reduced contrast enhancement^15–17^. However, most of these studies focused only on the automated detection of the IC. Only some of them investigated whether IC and PEN could be differentiated as well, which is technically more difficult as the differences in HU are less pronounced. In addition, these studies indicate that the HU differences of the infarct region compared to healthy brain parenchyma can be quite small in some cases and that, in particular, hardening artefacts near the skull bone make differentiation difficult^15–17^.

In this study, we investigated if spectral CT enables the detection and differentiation of IC and PEN in AIS based on monophasic CTA only. Virtual monoenergetic images at 70 keV by SDCT are considered equivalent to conventional images at 120 kVp from single-energy CT^18^. Accordingly, our results confirm that ischemic areas can be quantitatively distinguished from healthy tissue based on their density values at MonoE70^18,19^. However, like previous studies, our results also suggest that these differences can be quite small and may quickly reach borderline values. The focus of the present study was to evaluate the extent to which the application of SDCT offers new possibilities regarding this approach. SDCT enables the detection of material-specific properties and thus can precisely quantify and discriminate iodine-based contrast agent in tissue^6,8^. Hence, iodine imaging is an interesting technique for quantifying perfusion in contrast-enhanced CT examinations and has already been successfully applied to various clinical problems related to tissue perfusion, such as pulmonary artery embolism or mesenteric artery ischemia^11,20^. Moreover, SDCT provides spectral datasets for all protocols, which can be retrospectively reconstructed and post-processed. In contrast to that, other SCT-techniques such as dual-source or kV-switching do not provide spectral datasets with every scan, but specific protocols have to be chosen before scanning^7^.

We demonstrated the usefulness of SDCT for the detection of ischemic areas and the differentiation of IC and PEN using the different spectral parameters. The ischemic areas IC and PEN could be differentiated in all spectral maps based on the absolute values measured against the healthy parenchyma on the same side (HA) and against its healthy correlate on the opposite side (IC* and PEN*). Furthermore, the ratios calculated from the absolute values (IC/IC*, PEN/PEN* and HA/HA*) also displayed good detection and differentiation of ischemic areas. When comparing the individual spectral maps, the ID and MonoE40 maps showed the best detection and differentiation of the ischemic areas, both based on the absolute measurements and the ratio values. These results are also reflected in the visual assessment, as the infarct areas were best distinguished from the healthy areas in the ID and the MonoE40 map. The MonoE40 map close to the K-edge of iodine (33,2 keV) and the direct iodine quantification by the ID map best describe iodine accumulation in the tissue, which is the most important component reflecting perfusion. Increasing keV numbers move away from the K-edge and from accentuating iodine contrast^18,21^. Regarding ED, differentiation to healthy parenchyma is possible based on the absolute values measured, but, in relative terms, the distinction is significantly worse than with ID and MonoE40.

These results are consistent with those of previous studies on this topic. Hou et al. examined CTA in AIS patients and measured iodine concentration in areas of decreased cerebral blood volume and cerebral blood flow resembling the IC^22^. Like the present study, they demonstrated lower iodine concentrations in ischemic tissue than in healthy tissue. However, they did not investigate the distinguishability of IC from PEN, which is clinically highly relevant to guide treatment decisions. Furthermore, the authors did not investigate HU values in the areas at different keV levels^22^. In another study Fransson et al. showed that it is possible to distinguish the IC from the PEN based on contrast dynamics in multiphasic CTA examinations using the ID and MonoE40 maps^23^. However, it is important to consider that, although performing additional CTA phases does not imply additional contrast agent administration as in CTP, there is still more radiation than in monophasic CTA^23^. In the present study, only monophasic CTA examinations, as performed in multimodal CT for AIS imaging, were evaluated. Thereby, differentiation between IC and PEN seems possible already in monophasic CTA with the use of SDCT, which means less radiation exposure than with multiphasic CTA imaging.

In the clinical setting, the assessment of the IC and PEN and their proportionality is an important piece of information that needs to be assessed quickly as it influences therapeutic decisions^3,4^. As it takes additional time to perform the CTP, it would be beneficial to acquire these parameters from the CTA^1^. Based on our study results, SDCT demonstrates the potential to use monophasic CTA data to define IC and PEN in the clinical setting using predefined ratio values. For example, PEN/PEN* ratio are the lowest below 1 in ID, and cut-off values for defining PEN could be established, as achieved by using CTP analysis software^24^. In addition, the IC/IC* ratio is even lower than the PEN/PEN* ratio and cut-off values between the two ratios could also be defined to further differentiate between IC and PEN. Alternatively, in addition to the use of ratios, differentiation based on the absolute values measured on the infarct side alone, and thus independent from the healthy side, would also be conceivable. However, more extensive studies in general with a larger collective would be required to establish such cut-off values. Moreover, the improved visual delineation of the infarct area in MonoE40 and especially in ID maps showing, for example, the loss of a cortical iodine rim as a sign of severely hypoperfused cortex, already results in a potential application of these spectral maps in the clinical setting. Overall, the increasing importance and availability of SCT makes the technique described here highly interesting and reinforces the rationale for performing such a study survey. The results can probably also be applied to other SCT scanners (e.g., dual-source or kV-switch) but this would need to be investigated further. As another interesting approach, combined application with AI is also conceivable. The improved differentiability of IC, PEN and healthy parenchyma, and further artefact reduction could possibly improve AI-based post-processing and perfusion analysis^15–17^.

On the other hand, there are limitations that need to be named and discussed. A crucial methodological aspect is that in our study the semi-automated CTP analysis was used as a reference for the definition of the penumbra and infarct core areas and the further testing of the spectral maps was based on this. Formally, MRI with diffusion-weighted and T2-weighted sequences for the detection of cytotoxic and ionic oedema represents the gold standard in imaging of the penumbra and infarct core, and the question is raised as to whether this methodology would not be more suitable as a reference^25^. However, the problem is that MRI is rarely used in acute stroke imaging today due to problems such as limited 24/7 availability, delayed diagnosis, and higher costs^25,26^. Instead, multimodal CT including CTP is the clinical standard and has been analysed in numerous studies in comparison to MRI^3,12,27,28^. Although the study situation and guidelines show a formally higher sensitivity of MRI in diagnosis, CTP is established as a precise diagnostic method in stroke diagnosis and is therefore recommended^28,29^. Based on this study situation and the lack of MRI data in this retrospective study, the use of CTP was considered justified. In future prospective studies, however, the availability of MRI should be discussed and considered to optimise the methodological approach.

Another aspect concerns the exclusion criteria and the associated selection of the study cohort. The aim of this feasibility study was to investigate whether spectral analysis of CTA can detect and differentiate between penumbra and infarct core. For this reason, a sample was chosen that was as free from confounding factors as possible and that was precisely suited to the research objective. In order to avoid false CTP analysis being used for reference, patients with pronounced CTP artefacts, such as those caused by movement during the examination or insufficient contrast dynamics, were excluded. Such artefacts were thereby detected and indicated by the manufacturer’s semi-automated analysis software and the patients were excluded according to it. In fact, both CTP and MRI are susceptible to such artefacts due to their long acquisition times, so special attention must be paid to the avoidance of such artefacts in future studies.

Furthermore, a part of the initial cohort was excluded because they showed only a penumbra and no infarct core in the CTP analysis, or the infarct core indicated was very small (≤ 10 ml). As described above, the study’s aim was to evaluate whether the spectral analysis of CTA can detect and differentiate the penumbra and infarct core. In the author group’s opinion, the most suitable patient population for this purpose was one that also exhibited these two components to a sufficient degree in the CTP-analysis and allowed the analysis to be performed in accordance with the respective research question. Patients without evidence of a relevant infarct core therefore appeared to be less suitable for the research question and were therefore excluded from the present feasibility study in favour of a homogeneous, focused study cohort. Nevertheless, these patients represent a relevant group in clinical reality, and it is recommended that they should be explicitly addressed in future studies as part of the method’s further validation.

Other limitations concern patient-specific and technical aspects that can have an influence on tissue perfusion and contrast agent accumulation and should therefore be considered. For example, arterial contrast dynamics in CTA examinations may be suboptimal due to reduced cardiovascular status or too early venous contrast overlay. Furthermore, stenoses in the ascending cervical arteries must also be considered, as they can have a relevant influence on the dynamics of contrast agent accumulation in the infarct area compared to the healthy hemisphere. Finally, it should also be considered that the contrast agent dynamics and enhancement probably also depend on technical aspects of the examination protocol, such as the volume of contrast agent applied, the injection speed or the scan delay. In our study, the density values in the contralateral intracranial ICA and in the superior sagittal sinus were also measured in each patient, whereby the density in the artery was at least always higher than in the sinus. However, it remains unclear whether excessive venous contrast could have a relevant influence on the results of the analysis. Future studies should, therefore, also address these aspects to further validate the method and establish it for potential use in routine clinical practice.

## Conclusion

In conclusion, spectral imaging, and analysis by SDCT can reliably detect and differentiate IC and PEN in patients with AIS based on monophasic CTA data only and in comparison, to CTP. Hereby, detection and differentiation of IC, PEN and healthy tissue are best possible in the ID and the MonoE40 maps. Accordingly, this represents an interesting approach with potential benefit in clinical settings, saving time, contrast agent and radiation exposure.

## Data Availability

The datasets generated during and/or analysed during the current study are available from the corresponding author on reasonable request.

## Abbreviations

CT: Computed tomography
AIS: Acute ischemic stroke
NCCT: Native cranial computed tomography
CTA: Computed tomographic angiography
CTP: Computed tomographic perfusion
IC: Infarct core
PEN: Penumbra
SDCT: Spectral detector computed tomography
SCT: Spectral computed tomography
MonoE: Virtual monoenergetic
ID: Iodine density
ICA: Internal carotid artery
CCA: Common carotid artery
ISP: IntelliSpace portal^®^
ED: Electron density
ROI: Region of interest
HA: Healthy area
IC*: Corresponding area of the IC in the contralateral healthy hemisphere
PEN*: Corresponding area of the PEN in the contralateral healthy hemisphere
HA*: Corresponding area of the HA in the contralateral healthy hemisphere
HU: Hounsfield units
ROC: Receiver-operating-curve
AUC: Area-under-the-curve
SD: Standard deviation
ASPECTS: Alberta-stroke-program-early-CT-score
MRI: Magnetic resonance imaging
AI: Artificial intelligence
